# The pillars determining financial inclusion among SMEs in Egypt: service awareness, access and usage metrics and macroeconomic policies

**DOI:** 10.1186/s43093-021-00073-w

**Published:** 2021-08-04

**Authors:** Mohamed Samy ElDeeb, Yasser Tawfik Halim, Esmat Mostafa Kamel

**Affiliations:** 1 Faculty of Management Sciences, October University for Modern Sciences and Arts, 4/14 Zahraa Elmaadi, PO Box 11742, Cairo, Egypt; 2 Faculty of Management Sciences, October University for Modern Sciences and Arts, 99 A Road 9 Maadi, PO Box 11728, Cairo, Cairo, Egypt; 3 Faculty of Management Sciences, 6th of October, October University for Modern Sciences and Arts, Cairo, Egypt

**Keywords:** Financial inclusion, SMEs, Usage of banking services, Marketing awareness tools, Macroeconomic risk, Principal component analysis, Egypt

## Abstract

Over the past decade, financial inclusion has been a trending topic and key priority in developing countries seeking to build a resilient financial sector and pursuing economic growth. Most of the recently launched financial inclusion initiatives in Egypt, especially those aligned with the 2030 sustainability strategy, have targeted marginalized and excluded individuals. Only a few have addressed the financial inclusion of small- and medium-sized enterprises (SMEs). Accordingly, this paper aims to identify the main pillars of financial inclusion for SMEs. In keeping up with the mainstream literature, it introduces a number of financial inclusion determinants designed to attract SMEs. They include supply-side determinants such as access to financial services and marketing awareness campaigns, which act as tools to segment financial services and market their benefits to SMEs, and demand-side determinants, which involve the use of financial services. Finally, there is an assessment of the macroeconomic risks to investors and SMEs. The researchers’ methodology was based on first deriving a novel dataset from responses to a questionnaire addressing bankers who manage SME portfolios, second analyzing the dataset through descriptive and inferential statistics and third undertaking a twofold econometric estimation. The econometric estimations started with principal component analysis (PCA) and proceeded to a logistic regression to determine the significant variables pertinent to increasing the financial inclusion of SMEs. The PCA suggested three main pillars determining financial inclusion. They are integrated marketing tools, which increase SMEs’ awareness of and access to the most sophisticated banking services, usage of banking services, and assessment of the macroeconomic risks that would prevent SMEs from gaining access to financial services. As well, the interaction term between the variables derived from the three pillars accounts for a variability of 86.6% in the level of financial inclusion of Egypt’s SMEs.

## Introduction

Financial inclusion has achieved great notoriety among recently introduced concepts, especially after the onset of the 2008 global financial crisis. That crisis compelled international financial institutions to devise innovative strategies to achieve financial stability and provide access to the highest number of banking services possible. Once financial inclusion and stability are achieved, economic growth will be stimulated, and size of the underground economy will diminish. Many countries, especially developing ones, have derived universal policies from the World Bank Global Findex to achieve financial inclusion. Such policies have sought to introduce as many individuals/SMEs^1^ as possible to a scope of financial services and educate them about banking products and the macro-financial environment, with a view towards matching their capacities to the best alternative financial vehicles and preventing their financial marginalization. The policies have had a great impact on the economic and social conditions of individuals and SMEs alike on a national level [12, 25, 54].

When analyzing from the policy implication perspective, this topic would prompt policymakers to take actions and formulate their monetary policies on the basis of financial inclusion across countries. In turn, affecting the legal framework, the infrastructure of the financial markets, raising financial awareness, education and guaranteeing the protection of lenders' and borrowers’ rights, would realize the positive outcomes of financial inclusion. In essence, financial inclusion strategies have been defined as national or subnational roadmaps devised by stakeholders who contribute to their efforts and resources in achieving the objectives of financial inclusion. A financial inclusion strategy should address, for instance, the various problems related to financial services, the upgrading and development of the financial markets. It should also be sensitive towards the integration of financially excluded groups and informal sectors, entrepreneurs in general and businesswomen in particular [52].

Egypt has adopted steadfast measures to become a leader in the field of digital payments. It has worked toward launching a new phase of financial inclusion to meet its international sustainable development goals (SDGs) and carry out with its 2030 strategy. SDGs have focused on the Egyptian financial system, as one of the dynamos of economic growth. As well, reforms were applied in the financial sector to pave the way toward a smoother transition into the digital economy and to achieve an optimum level of financial inclusion. Above all, the new design of the financial system seeks to weave the transactions of individuals and enterprises into the country’s formal economic fabric and ensure that the development of financial institutions will provide them with a wider range of tailored services. It should also help to raise Egypt’s standard of living, narrow income disparities and reduce poverty rates by reaching out to the less-served segments of the society, such as the poor, the low-income and small- and medium-sized investors and startups [6].

To build a comprehensive financial system, there are multidimensional roles for the regulators of financial markets, including the Central Bank of Egypt, the Ministry of Finance and regulators of non-banking financial services. The first task for the regulators of financial markets is to create a profitable legal and regulatory framework: one that enables financial institutions to facilitate access to financial services for families and small businesses. Their second task is to reduce market imperfections and enhance market infrastructure by offering convenient payment channels and reducing transaction costs. Their third task is to recommend government intervention to help financial institutions build up the capacity of financial intermediaries, which increase awareness and reduce financial illiteracy among individuals and SMEs [28, 39]. Many benefits accrue when all stakeholders take part in building a resilient financial system based on regulations, infrastructural preparedness and awareness [25]. Indeed, financial inclusion will benefit both the public and the private sectors on both macro- and microeconomic levels. On the governmental front, financial inclusion has offered various social welfare programs that reached out financially to exclude beneficiaries. It has supported the Ministry of Social Solidarity to build reliable databases for beneficiaries and estimate income disparities [6]. A comprehensive financial system can help attract new businesses to the formal private sector, and especially to secure payment systems for day-to-day operations, improve services for employees and develop a variety of financial instruments to help women become entrepreneurs.

Finally, financial inclusion is already the cornerstone of any resilient financial sector; however, the criteria and measures for its definition remain subject to debate. Usually, policymakers adjust how financial inclusion can be adopted based on their needs and demographics of each economy. The existing literature has considered the experience of countries and agreed that the notion is undergirded by a number of pillars. On the supply side, financial inclusion is a matter of increasing access to financial services through the adoption of full-fledged awareness campaigns tailored to SMEs. On the demand side, it is based on criteria and key performance indicators that measure the usage of financial services by SMEs. There is also a macroeconomic dimension to the assessment of factors influencing SMEs' inclusiveness into the financial system [3, 27, 44, 60].

## Methods

Accordingly, the main objective of this research is to use primary research and a novel database aimed at SMEs in Egypt to identify non-traditional channels toward financial inclusion. We have tested the paper’s main objectives and hypotheses using a twofold methodology. First, we derived a novel qualitative dataset from a questionnaire distributed online to Egyptian financial banking executives who manage SMEs portfolios. The questionnaire used more than 42 variables to test for financial inclusion determinants. One of the questionnaire's advantages is that it can be adapted to a wider regional or international scale. The questionnaire response rate was 82%. Second, we undertook inferential and econometric modeling on the questionnaire, using both principal component analysis (PCA) and logistic regression to identify significant variables and reduce the dimension of unnecessary determinants of financial inclusion and test their robustness through the regression.

The PCA and regression results indicated that the interaction between variables such as SME access to the most sophisticated banking services through marketing, increased usage of tailored and digital banking services and assessment of macroeconomic risks pertinent to SMEs led to an 86.6% variability in the level of financial inclusion for Egypt’s SMEs. The paper is structured as follows: “Methods” section reviews the literature, “Literature review” section lays out the questionnaire’s data design, “Exploratory research prior to questionnaire design” section frames our hypotheses, “Methodological approach: twofold approach” section describes our methodologies, “Results and discussion” section presents and discusses main results and defines the limitations, and finally, “Conclusion” section concludes.

## Literature review

### Role of financial inclusion in promoting societal segments and, above all, SMEs

The literature has settled on a broad definition of financial inclusion, but it has also adopted a wide variety of perspectives to explain the term from different angles and to differentiate between individuals, businesses and stakeholders needs for financial inclusion. The World Bank’s Universal Financial Access, the IFC 2020 goals and the Alliance for Financial Inclusion all agree that financial inclusion is in a nutshell, the universal availability and usage of high-quality transactions and payment products tailored to different segments of the society and to firms of different sizes. The Financial Inclusion Center in Washington complemented the previous definition by adding that individuals and businesses are not excluded, if they are introduced to the most innovative approaches that will guarantee their access to a full range of suitable financial services at affordable prices [12, 54, 61]. Hence, the cost of financial services is one of the crucial elements in determining financial inclusion. Accordingly, this cost has fallen dramatically and amplified the access of remotely located or vulnerable individuals and women to financial services. Additional banking policies have worked in a highly effective manner to offer duty-free accounts, facilitate and unify the multiple documentation requirements for applicants and accelerate the use of electronic transactions through bank accounts and bank wallet accounts with some of them tailored to the individuals and others to SMEs needs [28].

Small- and medium-sized enterprises (SMEs) can be defined, as the workhorse of economic growth since they drive employment through job creation. In fact, their contribution has reached up to 50% of GDP in some countries [34, 42]. The greater an SME’s access to finance, the wider the range of financial services available to jump start its business and expand its operations [12, 54, 61]. SMEs account for 45% of total employment globally and for up to 70% of jobs in most OECD countries [7]. More importantly, the investments of SMEs are directly channeled to promote innovative services and accommodate to the expansive digital revolution in the financial sector [35]. Finally, to qualify for microfinance services, SMEs should follow a set of financial regulations and requirements set by banks and non-financial institutions. Based on OECD definition and classification for SMEs, a small-sized enterprise is characterized by the number of employees that should not exceed 10–49 employees and their balance sheets of EUR 10 million, whereas for a medium-sized enterprise the range is from 50 to 249 employees and their balance sheets of EUR 50 million [43].

### The literature’s determinants of financial inclusion for SMEs

One stream of the literature advocates the removal of price and non-price obstacles to facilitate SMEs' access and usage of financial services and ease common barriers across countries, which will facilitate SMEs growth. There is a consensus in the mainstream literature on three standard factors working on the acceleration financial inclusion. The first is the availability and affordability of formal, electronic and secure financial services tailored to the needs of SMEs. The second is the need to integrate the most vulnerable investors and startups into the formal financial fabric, which can be done solely through full-fledged promotional campaigns and the spread of financial literacy [49, 56]. The case of Dahmen and Rodriguez [18] has shown how in Colombia and many other countries, financial literacy was positively correlated with the promotion of bank account use and the facilitation of financial payments for firms.

The third is the necessity of providing financial services on the basis of SMEs’ performance ratios. Under some circumstances, small-sized enterprises can be given credit against a guaranteed collateral and the existence of highly valued fixed assets. The lower a firm’s ownership shares in tangible and non-tangible assets, the less likely it is to be approved for external credit lines, regardless of its demographic characteristics. Fourth, a firm’s age^2^ is linked to its reputation, historical performance, ability to handle financial distress and the ability to build up trustworthy credit records that are essential for the assessment of its credit scores [40, 46]. A firm’s business activities and performance are used as indicators to determine its access to credit, loans and mobilize financial payments and transfers. Firms in the construction sector, for example, have higher guaranteed collaterals due to the size of their fixed assets. In turn, these assets would generate higher profitability ratios and consequently increase their access to more funds.

Fifth, and last, the presence of macroeconomic and financial risks embedded in the economy is critical to SMEs’ financial inclusion. The macroeconomic risk to which SMEs are exposed is highly dependent upon the central bank’s directives and monetary policy that opts to set the appropriate interest rates. Usually, optimal interest rates set by central banks seek to generate higher output while curbing inflation trends and economic volatility [38]. The literature motivated by Evans and Adeoye [22] has indicated to the causality running between financial inclusion and the effectiveness of monetary policies, especially for firms in developing and African economies. This is one of the limitations in this branch of the literature, as it is not exactly known whether financial inclusion will affect and alter monetary policy targets or if the adoption of monetary directives might invigorate financial inclusion efforts.

### Obstacles preventing SME access to financial services

Historically, many obstacles have hindered SMEs’ access to financial services, particularly in developing countries. They include limited spread of knowledge and illiteracy, which could prevent the use of the most suitable financial and digital services. As well, the lack of knowledge tends to block proper decision making with respect to the business financial affairs and also undermines the importance of the most up-to-date and suitable online banking services. Additional obstacles include higher transaction costs, red tape and the excessive documentation required to open and close accounts. These factors have already relegated about 35% of SMEs from being financially included [18, 31, 42, 61]. The MSME survey diagnostics elaborated by Clarke et al. [17] has identified the fragility of data protection laws, as yet another problem eroding off SMEs confidence in the financial system. Finally, Karpowicz [31] has illustrated that the depth of financial services that banks provide to SMEs is determined by the collateral, guarantees and credit lines taken in return, which are sometimes too strict and hinder their growth. At the same time, the interest rate they receive when evaluated is still insufficient compared to the higher transaction costs they incur. SMEs in developing countries are therefore in dire need of bridging the ‘financial gap’ between demand for the amount, variety, quality and means of financial services and the awareness of the payment services offered.

### Financial inclusion through digitalized banking services: the case of Egypt’s SMEs

Financial inclusion has been one of Egypt’s national priorities since the launch of its Sustainable Development Strategy (SDS) Vision 2030, but by then the country had already been taking steps to transform its economy into a digital one reliant on e-payments and reducing the use of cash. The Central Bank of Egypt (CBE) has participated in several regional and global initiatives to improve financial inclusion. In July 2017, for example, Egypt was chosen along with China and Mexico to be a model country in the Financial Inclusion Global Initiative launched by the World Bank Group. The three-year initiative aimed to support access to financial services for the unbanked and underbanked and developed policy recommendations for digital finance. According to the World Bank, Egypt had the potential to bring a large number of entities and individuals estimated at more than 44 million adults into the formal financial sector [53]. The latest banking statistics for 2018 indicated that there are more than 2,800 branches for 39 separate banks distributed across governorates. Almost half of these rely mainly on e-banking and mobile banking services, with 133,651 registered payment agents to provide a variety of electronic financial services to individuals and firms [14]. The Egyptian financial sector has revamped its strategy along two main axes: First, it has updated the regulatory framework to suit new financial products introduced onto the market and to safeguard firms against cyber fraud; second, it has stimulated digital transactions and broadened e-payment channels to include a variety of customized services for SMEs. Among those services, fall the mobile transaction modes such as cardless ATM, cash-in/cash-out for mobile wallets, person-to-person (P2P), person-to-merchant (P2M), merchant-to-merchant (M2M), international money transfers (IMT), virtual card number (VCN) and bank wallet accounts [30, 48].

### The main pillars activating financial inclusion in the case of Egypt’s SMEs

#### Financial services access and usage metrics

Banks are mainly using sales volume as a metric to grant access to specific financial services and to distinguish between services offered to corporates and others to SMEs. Classifying a business as an SME is in fact inconsistent across countries, and there is no one standardized definition for an SME. An SME might be considered of medium size in one country and can again be classified as a large-sized enterprise elsewhere. Countries pursuing financial inclusion must therefore carefully define their business size and characteristics to match the procedures and services offered to different categories of firms through formal financial institutions. The banking sector of many developing countries has started to cater to the needs of SMEs through offering a variety of financial services, such as financing and saving plans. These plans incorporate risk mitigation strategies for SMEs into their design to guarantee an amplitude of services and low-cost transactions [28, 53].

In fact, the spread of technology in the form of digital e-payments and mobile payment services is one of the main facilitators of access to financial services for small investors and businesses and it is effective in lowering both costs and risks. Account penetration in Egypt increased from 10 to 33% from 2011 to 2017 due to the support of cashless transactions and banks’ own encouragement of individuals and businesses to open accounts at lower fees and lower restrictions on cash deposit requirements. Moreover, the Central Bank of Egypt (CBE) has been a member of the international alliance for financial inclusion since 2013, which represented a great opportunity for hands on practical and technical know-how to stress on the concept of financial inclusion among businesses and startups [11].

The CBE has encouraged banks to expand their financial services for SMEs and increase the proportion of their total lending that goes to SMEs by at least 20%. They have also encouraged private and public banks to establish special units devoted to providing SMEs with financial services. In light of those directives, some 49 billion Egyptian pounds went toward financing SMEs during 2017. To accelerate SMEs growth and activities, many incentives were set up, such as reductions in documentation and procedures for account opening, mobile and online access to banking services and consistent monitoring of financial services [28]. Easier access to financial services in turn mobilized household savings and freed up capital to finance SMEs. It offered financial services on an equitable basis to citizens and SMEs that had otherwise been excluded for decades.

#### Raising financial literacy and awareness: marketing campaigns as an outreach to SMEs

The modern banking system is under continuous pressure from the shocks and influences of exogenous and economic risks. The explosive growth of new trends in digital technologies has exacerbated for banks the challenge of coping with the ever-increasing expectations of their customers. They had to innovate to meet the ‘new’ needs and wants of SMEs as a target group [23, 58]. According to Fortea and Ioan [24], only a few of the branded big banks have used direct marketing to increase SMEs’ awareness of their services. Some have prioritized promotional activities, such as sweepstakes for credit cards. Most have engaged in public relations (PR) to strengthen their credibility and alter the perception of their corporate image. At any rate, it has represented a small portion of their marketing budget.

Direct marketing campaigns held by banks are mainly focused on telemarketing and the Internet, which consume only a small portion of the total marketing budgets allocated to SMEs. To evaluate the effectiveness of each marketing medium used, we must screen and select the best mix of cost-effective marketing tools. Those tools should reduce financial illiteracy and, at the same time, deliver a clear, consistent message through all possible communication channels [33, 47, 51]. Marketing communications tools should be focused on the following four components: first, establishing a comprehensive advertising campaign to promote a bank’s credibility, reputation and corporate image to SMEs; second, promoting new and modified products to be aligned with external conditions and internal business strategies; third, engaging in permanent public relations, regular interviews and media events, so as to inform SMEs of a bank’s regular activities; and finally, upscaling employees' skills and executive knowledge, as they leave the greatest impression on the SME perception of a bank’s financial products, services and their quality.

Claessens and Laeven [16] pinpointed that many new trends over the past few years have changed the dynamics of the banking industry, especially in Egypt. The trends include globalization, regulations and deregulations, the opening up of domestic markets, improvements in retail banking, the building of corporate image, the launch of new brands, the opening of new delivery channels and, finally, the pursuit of smaller connections to SME customers. International trends in the banking sector have moved beyond the traditional means of conducting transactions. Most national and international banking sectors have started to rely on and promote digital banking tools. In fact, the emergence of Internet banking in Egypt has sustained an effective two-way interactive communication between banks and SMEs through an advertising budget tailored to customers. It has also increased the number of non-financial institutions that act as mediators to grant access to financial services, especially during the latest COVID-19 pandemic. Banque Misr, the National Bank of Egypt, as well as Monzo Bank Ltd. in the UK and Enpara.com digital Bank in Turkey, have moved to fully digitalize their services on the domestic level during the pandemic. It is important to train the bank’s staff to deal with and communicate easily to customers. Also, banks should be capable of tailoring and marketing financial services to be compatible to the demographic structure of SMEs [20, 26, 37, 55].

#### Macroeconomic and financial risks to SMEs

The theoretical and empirical literature has covered all aspects of financial inclusion, which is believed to accelerate economic growth. This strand of the literature suggested links between macroeconomic indicators and financial risk; in addition, it has gauged the impact of these links on SMEs’ financial inclusion and vice versa. Previous studies used GDP per capita, per capita income, consumption per capita and the informal sector as a percentage of total GDP to estimate the financial power and resilience of individuals and businesses. There are, besides, other key indicators such as inflation volatility, output growth and income disparities used to construct the Gini index, which acts as a proxy for measuring income equalities [32, 41, 44, 60]. The higher percentage of credit allocated to a startup would lead to higher generated income, which would in turn accelerate economic growth and reduce income inequality. One of the obstacles impeding the financial inclusion of firms was attributed to the higher transaction costs recorded in less developed neighborhoods. Consequently, the overstated transaction costs were not suitable for Egypt, which is characterized by higher-income inequalities across governorates [3, 27, 44, 60]. Other empirical models have predicted that causality runs in the opposite direction between income inequality and financial inclusion. Perotti et al. [45] found that countries with less inequality were well prepared for greater access to financial services.

The implications of financial inclusion on the economy and how it promotes economic growth are multidimensional, especially when it comes to minimizing the size of the shadow economy and mitigating tax evasion [62]. They are best identified by examining the relationship between financial inclusion and its broader dynamics on an array of macroeconomic indicators. For example, a consistent inflation rate could be achieved through an optimal monetary policy, with an interest rate set to withdraw excess liquidity from an expanding market or inject it into a contracting one. This optimal policy mitigates severe liquidity shocks by introducing a variety of financial products segmented to SMEs. Directing services at SMEs should in turn moderate per capita consumption and induce additional investments into SMEs through access to loans and financial products. This should lead to output stability, higher disposable income per capita, higher per capita GDP and less tax evasion among SMEs and other firms [32, 41, 44, 60].

## Exploratory research prior to questionnaire design

Researchers began with an exploratory study of what bankers at their various managerial levels recommend for the design of variables to determine the financial inclusion of SMEs. The exploratory study was conducted through personal interviews with managers and decision makers concerned with the research area in the private and public banking sector under study. A random sampling technique was used by researchers, and it included bankers at managerial and non-managerial positions in seven banks in Greater Cairo. The results were fed into the second part of the data collection, which was reflected in the design of a questionnaire covering all aspects of financial inclusion for SMEs. The determinants of financial inclusion were derived from the primary data obtained through the online questionnaire.

### Questionnaire design and data sampling

The questionnaire consisted of five sets of questions. The first covered the multiple saving services guaranteed to SMEs. The second measured the effectiveness of the marketing tools used to increase SMEs awareness about the services. The third evaluated the usage and metrics of financial services provided to SMEs. The fourth assessed the macroeconomic risks SMEs are exposed to and how it might impact their inclusion into the financial services. Finally, the fifth covered demographic factors, such as the degree of their access and integration to financial inclusion. The questionnaire consisted of 42 close-ended questions. Thirty-eight questions were designed on the basis of a categorical Likert scale and the rest following a nominal scale. Additional data were collected on the respondents themselves, such as gender, years of work experience, type of bank and educational level to shed light on the role of bankers on influencing SMEs decisions. The response rate was 82% to represent 355 submitted questionnaires out of 432 questionnaires.

**Fig. 1:**
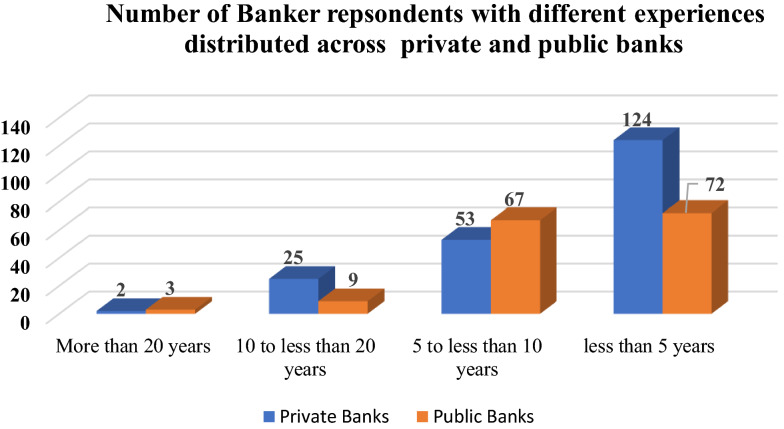
Cross-tabulation between bankers' respondents' years of experience and type of bank. *Data Source*: questionnaire enclosed in “Appendix”

The researchers used a practical, non-probability sampling technique, called accidental sampling, to maximize accessibility, time saving and keep up with coding the collected data. They submitted the questionnaires online, through professional social media platforms such as LinkedIn to reach to as many of the bankers working in the Egyptian private and public banks. As shown in Table 1 and Fig. 1, the sample was almost evenly distributed between female (48.2%) and male (51.8%) respondents. Almost (55%) of respondents had less than five years of experience, (34%) had more than five and less than ten years of experience, and a minority of 9.6% worked for more than ten years. A majority (58%) of respondents worked for private banks and most of them had completed more than 5 years of experience and (42%) in public banks. All of the banks served Egyptian SMEs.

**Table 1:** Summary of frequency statistics for the variables *Data source*: questionnaire enclosed in “Appendix”

Indictors	Frequency	Percent	Valid percent	Cumulative percent
*Respondents gender*				
Female	171	48.2	48.2	48.2
Male	184	51.8	51.8	100.0
*Years of experience at work*				
10 to less than 20 years	34	9.6	9.6	9.6
5 to less than 10 years	120	33.8	33.8	43.4
Less than 5 years	196	55.2	55.2	98.6
More than 20 years	5	1.4	1.4	100
*Type of bank*				
Employees in private banks	205	57.8	57.8	57.8
Employees in public banks	150	42.2	42.2	100

**Fig. 2:**
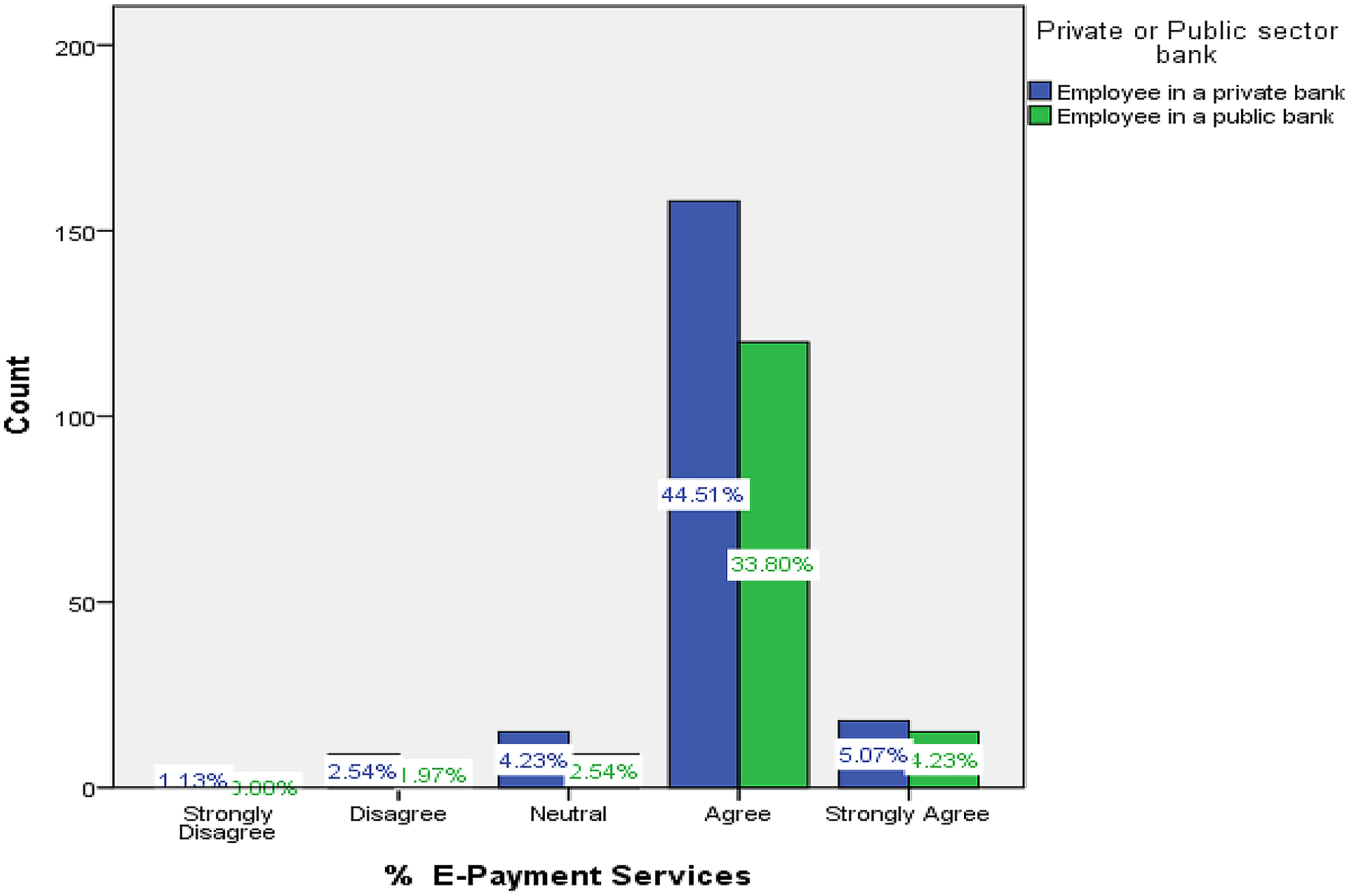
Breakdown of access to e-payment services provided to SMEs by type of bank. *Data Source*: questionnaire enclosed in “Appendix”

The cross-tabulation in Fig. 2 for the variables of e-payment services and type of bank shows that around 78% of public and private banking respondents agreed that access to e-payments facilitated SMEs’ transactions, increased their interaction with formal financial agencies and enabled the inclusion of many more small-sized enterprises and startups in the financial system. The coded data derived from the questionnaire enclosed in “Appendix” has indicated that 75% of respondents believed that offering multiple saving services to SMEs would encourage them to join formal financial banks. In addition, a vast majority of 80% strongly agreed that loan procedures, their paperwork, collaterals and restrictions should be designed to facilitate SMEs operations and their access to funds. As for integrated marketing campaigns, 33% of respondents were neutral and they did not agree to the efficacy of promoting financial services to SMEs through enhanced direct sales and promotional tools; meanwhile, a majority of almost 87% of bankers serving to SMEs believed that both advertising and PR campaigns provided more know-how on all types of financial services. In addition, 72% agreed that Internet and social media campaigns directed at SMEs were one of the most highly effective marketing and communication tools.

The coded data derived from the questionnaire in “Appendix” illustrated that there was a conflict between respondents on the idea of having SMEs pay higher fees and transaction costs. Some 32.7% were neutral or did not confirm whether banks charged SMEs a high percentage of fees per transaction, which would act as a barrier against financial inclusion, whereas 40% of private banks agreed that SMEs were charged a high fee per transaction. As well, more than half of the respondents believed that the growing volume of transactions for SMEs was highly related to their higher net income. Moreover, respondents had a strong sense that the central banks’ macroeconomic policy played a critical role in stabilizing the financial sector, as 63% strongly agreed that its monetary policies restored SMEs confidence in the effectiveness and credibility of the banking system and prompted them to open more accounts and join formal financial institutions and agencies.

More importantly, around 86% indicated that SMEs evaded their taxes due to the high and uneven tax levels, when they reported their net income and this in turn diminished their operations. As previously mentioned in the literature, although SMEs with outstanding loans are seen as the engine of financial inclusion and for Middle East, North Africa, Afghanistan, and Pakistan(MENAP) countries, only the average share of SMEs in total bank lending stood recently at a modest 7% [12]. In consistency with the literature data, more than 50% of respondents believed that the number of SMEs with outstanding loans from regulated or formal financial institutions should increase to open the door to SMEs to access more funds and start operating under sustainable conditions.

### Research framework and hypotheses development

#### Main hypothesis to be tested and applied to questionnaire design

On the basis of the above illustrated literature, four hypotheses were developed. Figure 2 in “Appendix” illustrates the framework analysis:

##### **H1**

The following are the most significant determinants of financial inclusion: -

##### **H1a**

The higher levels at which SMEs may access multiple segmented savings and deposit services, the higher will their level of financial inclusion be.

##### **H1b**

The more instructive the integrated marketing tools used to raise awareness about the services provided to SMEs, the higher will their access to financial services and the level of financial inclusion be.

##### **H1c**

The more practical will the financial services offered by banks to SMEs', the higher will their access to financial services and the level of financial inclusion be.

##### **H1d**

The higher the interest rate set through the optimum monetary policy, the lower will SMEs be able to access loans and the lower their level of financial inclusion.

## Methodological approach: twofold approach

### Methodological design

The paper’s methodology followed a twofold approach. First step, novel primary data were obtained from the online questionnaires to be consistent to other authors using primary data in determining factors influencing financial inclusion (see [12, 19, 50]). As previously mentioned, the questionnaire addressed private and public-sector bankers in managerial and non-managerial positions serving to SMEs. Second, the collected data from the questionnaires were subject to descriptive and inferential tests (summarized in “Methods” section). The inferential estimations undertaken focused on the principal component analysis (PCA). As previously mentioned, the dependent variable to be estimated was the level of financial inclusion based on a number of indicators^3^ included in the questionnaire. This variable is a categorical variable estimated as the percentage of SMEs with accounts, outstanding loans, payroll accounts and access to e-banking services. After the analysis of the coded answers, a set of 42 independent variables were derived from the questionnaires to determine the level of financial inclusion (see “Appendix” questionnaire close- ended question).

It was theoretically and empirically impractical to introduce the 42 independent variables into a classical regression. Therefore, it was inevitable for the researchers to reduce the number of variables through the principal component analysis (PCA). The PCA is an inferential analysis method used to summarize the number of variables determining in this case SMEs’ level of financial inclusion, and its use is aligned with the previous literature using the same approach, as in the case of Tomaselli et al. [59], ElSherif [21], Bensaada and Taghezout [10] and Cámara and Tuesta [13] to estimate the level of financial inclusion. After the PCA, a logistic regression with categorical variables was conducted to provide the best fit to a linear regression model on the factors causing the highest possible variability on the level of financial inclusion (as proposed by Archer et al. [4]). The advantage of a logistic regression is that it accounts for more than one category of both the dependent and independent variables through the maximum likelihood estimation and works well with interval and categorical data [2].

### Principal component analysis (PCA) to determine SMEs’ level of financial inclusion

The rest of this section will be devoted to an inferential estimation technique known as PCA, which served as a preparatory step to the regression analysis. Shlens [57] defined PCA as a statistical approach that examines interrelations between a set of variables and identifies the underlying structure of those variables. It generates one or more artificial series, summarizing the behavior of a group of variables. It is computed in such a way that the first three or more components under which variables will be loaded should explain as much as possible of the variability in the results. It treats at the same time for multicollinearity and possible autocorrelation between underlying variables by grouping the variables into a smaller number of original components based on their original scores and mathematical properties, and these components are not linearly dependent [9]. An additional benefit to PCA, as noted by Cámara and Tuesta [13], lies in the new set of classified variables, which it generates to explain the changes in the dependent variable. These are not necessarily correlated but certainly hold and describe a big portion of the underlying latent structure of the variables introduced before the PCA.

### Logistic regression model analysis with ordinal variables

After extracting the principal components meant to determine variability in the level of financial inclusion, the main components PCA 1, 2, 3, 4, … driven from the inferential analysis will be fed as variables into a logistic regression estimation. This type of regression provides for more than one category of both the dependent and independent variables and is benchmarked in the technical literature, as a multinomial logistic regression (refer to Barbić et al. [8]). An interval logistic regression another similar estimation was used as a robustness check to suit the nature of the Likert-scale questions introduced earlier in the questionnaire design. Both types of regressions treat for multicollinearity, as a nonlinear relation exists between the variable and the intercept term. It is well known that multicollinearity might lead to errors in the estimation, resulting in linear dependency and less-consistent results. Hence, one of the virtues of multinomial logistic regression is its potency to resolve for the omission of variables and at the same time preserve the predictive power of the model.

The generalized equation for the multinomial logistic model for $\theta _{j}$, which denotes the level of financial inclusion measured through categories of the previously mentioned indicators and which varies by a single or more than one independent variable, is written as follows:-

${\text{Ln}}(\theta _{j} )~ = \propto _{j} - \beta X$… and j denotes number of categories to1, whereas $\propto _{j}$ is the intercept term. $\beta$ is the independent variable and it is expanded in its simplest form with more than one independent variable [36].

The ordinal and categorical logistic regression at its expanded version is as follows:

1 $${\text{Logit}}~\left( {P\left( {Y < = j} \right)} \right) = ~ < < _{{j0}} + _{{j1}} x_{1} + \cdots + _{{jp}} X_{p}.$$

## Results and discussion

### Main results for PCA

The PCA summarizes and reduces the size of the 42 independent variables into a number of underlying factors and structure, which determine the variability of financial inclusion for SMEs in Egypt. In keeping up with the literature (notably [1, 8, 10, 13]), the ‘eigenvalue-based criteria,’^4^ provided the cut-off threshold for the number of components or groups explaining the variability of the dependent variable. There is a standardized cut-off point agreed upon by the literature, which should exceed 50% or equivalent to a number of PC groups that retain a coefficient of variability that almost reaches one [29]. There is in addition an acceptable reference value produced by Cerny and Kaiser [15], which adopts Kaiser–Meyer–Olkin sampling adequacy criteria to check the adequacy of the sample and its underlying structure, the design of variables used and introduced into the PCA. The coefficient reference value needs to exceed a value of 0.5, so the coefficient obtained from sampling adequacy test stood at 0.8 with high levels of significance. This result derived was plausible, when compared to the reference value.

**Fig. 3:**
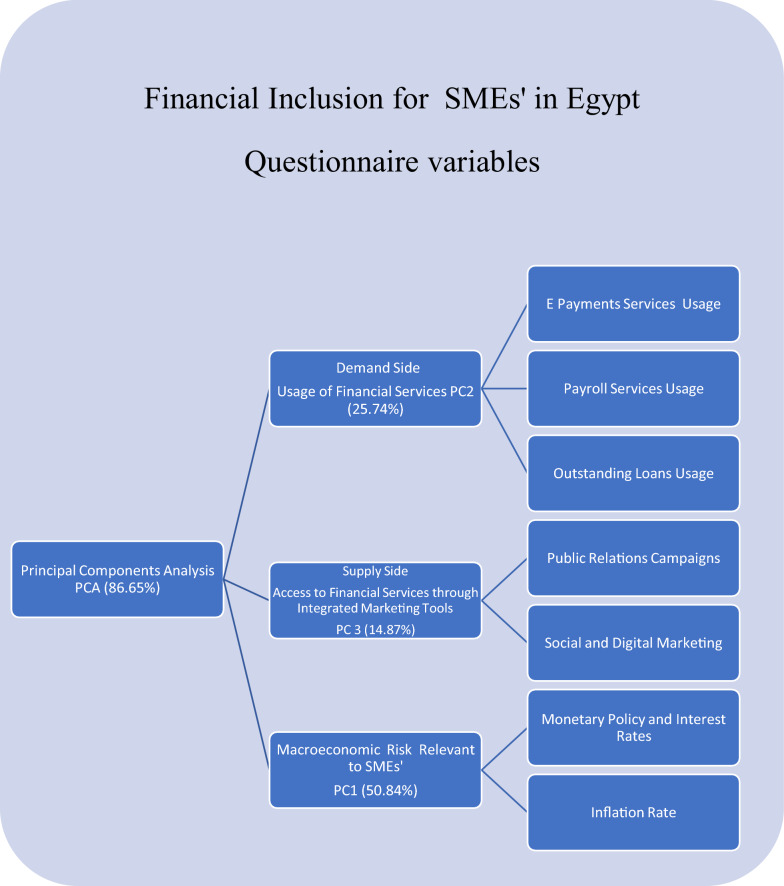
The pillars of financial inclusion based on hypotheses testing, questionnaire and PCA results. *Source*: Figure pillars collected through the questionnaire descriptive data

Later, to estimate the PCA, we used the ‘varimax method’ to rotate factors and reduce the number of variables that load on the principal component vectors or what will be called as the sub-factors loading on the main components and explaining the variability in financial inclusion. Tables 2, 3 and Fig. 3 in “Appendix” all refer to the PCA that resulted in three components with their uploaded variables. These explain 86.65% of the total cumulative variance and original data that determine the level of financial inclusion of Egypt’s SMEs. The peculiarity of the results is derived from the interaction between categories of several variables that account for the effective variability in SMEs’ level of financial inclusion.

**Table 2:** Total variance explained

Factor	Initial eigenvalues			Rotation sums of squared loadings		
	Total	% of variance	Cumulative %	Total	% of Variance	Cumulative %
1. Macroeconomic risk pertinent to SMEs)	4.559	56.982	56.982	2.607	50.847	50.847
2. Usage of financial services by SME's	1.299	16.238	73.220	2.459	25.745	76.7
3. Marketing promotion tools to SMEs	1.074	13.427	**86.647**	1.866	14.877	**86.647**
4. Type of SMEs sector [E1]	.324	4.051	90.698			
5. Account transaction costs for SMEs [C2]	.298	3.728	94.426			
6.Sales promotion SMEs [B1]	.164	2.052	96.478			
7. Tax evasion SMEs [D4]	.155	1.933	98.411			
8. Number of employees SMEs [D4]	.127	1.589	100.000			

The bold value indicates to the cumulative percentage pf 86.65% covering most of the variability of financial inclusion through the "three PC 1,2,3 and which represent:- PC1: Macreconomic risj pertinent to SMEs PC2: Usage of financial services by SMEs PC3: Marketing promotion tools to SMEs

**Table 3:** Total sub-factors loaded under each PC group

PCA 1 (macroeconomic risk pertinent to SMEs)	Explained variance	PCA 2 (usage of financial services by SMEs)	Explained variance	PCA 3 (marketing promotion tools to SMEs)	Explained variance
D1: monetary policy (Interest rate)	.884	F2: SMEs with outstanding loans	.860	B3: PR campaign	.864
D2: Inflation rate	.909	F3: SMEs with payroll accounts	.882	B4:Social media campaign	.889
Interaction between D1* D2	.914	Interaction F2* F3	.893	interaction between B3*B4	.891

Extraction method: principal component analysis. Rotation method: varimax with Kaiser normalization. A. Rotation converged in five iterations

The total variance is decomposed as follows. In Table 2, the first component, PCA 1, generated a 50.84% variability in financial inclusion and it reflects the macroeconomic and financial risk variables and their sub-factors such as optimal interest rate and inflation rate. PCA 2 generated a 25.74% variability and pertains to variables related to financial services usage among SMEs. These sub-factors include percentage of accounts used, loans from various sources, payroll accounts and e-payment services. PCA 3, representing greater access to financial services through integrated marketing campaigns, generated a 14.87% variability in the level of financial inclusion for SMEs. As for the sub-factors loading on the three PCA exhibited in Table 3 in the following manner. PC 1 of highest variability which is macroeconomic volatility and risk is determined by the setting of optimal interest rate and the inflation rate and interaction between them. PC2 pertinent to the usage of financial services includes the percentage of outstanding loans for SMEs and payroll accounts and their interaction, and finally PC3 causing the least variability of all components is comprised of PR and social media campaigns to market and raise awareness among SMEs and their combined effect led to the variability on the level of financial inclusion.

It is worth mentioning that all eigenvalues exceeded 1 and that a validity check of the results was performed through a reliability analysis called “Cronbach's alpha.” This gave us an estimate of 0.896, as indicated in Table 4. Here, Cronbach's alpha tests for high consistency in the original and latent variables are used to explain for financial inclusion. The results were aligned to similar values derived from the literature [8].

**Table 4:** Reliability statistics

Cronbach's alpha	Cronbach's alpha based on standardized items	No. of items
.896	.767	10

### Main results for logistic regression estimations

Once the principal components were extracted through factor analysis, the second step was to introduce a logistic regression with categorical variables to estimate the impact of PC1, which is the macroeconomic and financial risk. PC2 reflects the increased usage of banking services, and PC3 focuses on the access to services through full-fledged integrated marketing campaigns directed to raise the awareness of SMEs. Table 5 shows that the model's three PCs used to predict the level of financial inclusion are highly significant, with a confidence interval of 95%. The ‘goodness-of-fit’ model failed to reject the null hypothesis that the three PCA factors explain correctly the variability in financial inclusion of SMEs. Finally, Table 6 shows that the model’s coefficients reach their highest significant values for the use of financial services variables and macroeconomic risk at coefficients of 0.527 and 0.444, respectively. Finally, integrated marketing campaigns increased the visibility of and access to financial services for SMEs at a lower coefficient of 0.187 at high levels of significance.

**Table 5:** Model fitting information

Model	Model fitting criteria	Likelihood ratio tests		
	-2 Log likelihood	Chi-square	df	Sig.
Intercept only	869.939			
Final	608.997	260.943	12	.000

**Table 6:** Coefficients

	Standardized coefficients		df	F	Sig.
	Beta	Bootstrap (1000) estimate of std. error			
Macro-financial risk	.444	.056	4	62.729	.000
Usage of financial services	.527	.054	3	95.469	.000
Access through IMC	.187	.036	3	26.444	.000

### Some limitations in the methodological approach

It is essential at this point to explain some of the limitations of this paper. One of the first limitations that should be considered will be the absence of a standardized definition for an SME and more precisely to the banking community and the criteria upon which bankers would provide access to financial services targeted at SMEs. Based on OECD, a small- and medium-sized enterprise is defined as the enterprise; whose number of employees should not exceed 10–49 employees for small-sized enterprises and balance sheets of EUR 10 million. As for medium-sized enterprises, the range is from 50 to 29 employees and balance sheets of EUR 50 million [43]. The second limitation was particular to the causality running between macroeconomic stability and level of financial inclusion, which would have been totally resolved through using the propensity score matching (PSM) approach or through 2SLS to address this bias; however, it would have extended the work's scope to treat for this problematic bias elaborately.

## Conclusion

This paper contributes to the literature on financial inclusion in many ways. First, the researchers have provided a new perspective on the financial inclusion of SMEs and the expansion of the financial services provided to them in Egypt. The paper singles out financial literacy in particular and how it could be fostered through integrated marketing campaigns special tools such as social media and public relations aimed at SMEs. Second, it considers the macroeconomic risks and the role of optimal monetary policy in maintaining a consistent level of financial inclusion and mitigating inflationary trends that would harm SMEs. Third, driven from the literature, an online questionnaire was submitted to bankers who served SMEs and it was used to obtain a new dataset, which suggested actions that banks can take to increase the inclusion of SMEs into the official banking system. The results became robust after they were tested through inferential modeling applying the PCA and the econometric modeling using logistic regression.

The main three factors derived from PCA led to 86.65% of the variability in the level of SMEs' financial inclusion, and they were organized chronologically to include: PC1 which reflects on the understanding of the sensitivity of macroeconomic policies and how they support financial inclusion of SMEs; PC2 which refers to the usage of SMEs to loans and employees' payrolls; and finally, PC3 which targets the provision of higher access of SMEs to financial services through financial literacy promoted via integrated marketing campaigns and tools. In a nutshell, the questionnaires and the paper’s main results suggest to a promising line of research, which will investigate and test new metrics and factors to be accounted for and included on financial inclusion indices not only for the case of Egypt but on a regional and international scale. They also underscore the importance of e-payments and other online services, which can be prompted through innovative marketing tools and scaled to the needs of SMEs and their respective business operations. As well, this paper emphasized on the policy implications addressing bankers and drawing out on the most untraditional channels through which bankers could approach SMEs. It is known that in many developing and emerging countries, SMEs represent one of least financial included groups in their economies and at the same time they are considered the locomotive of economic growth. Thus, more services and follow-up should be dedicated to SMEs through financial and non-financial institutions and agencies to reinforce SMEs role as the workhorse of economic growth, generation of employment and creation of jobs.

## Figures and Tables

**Table 8:**
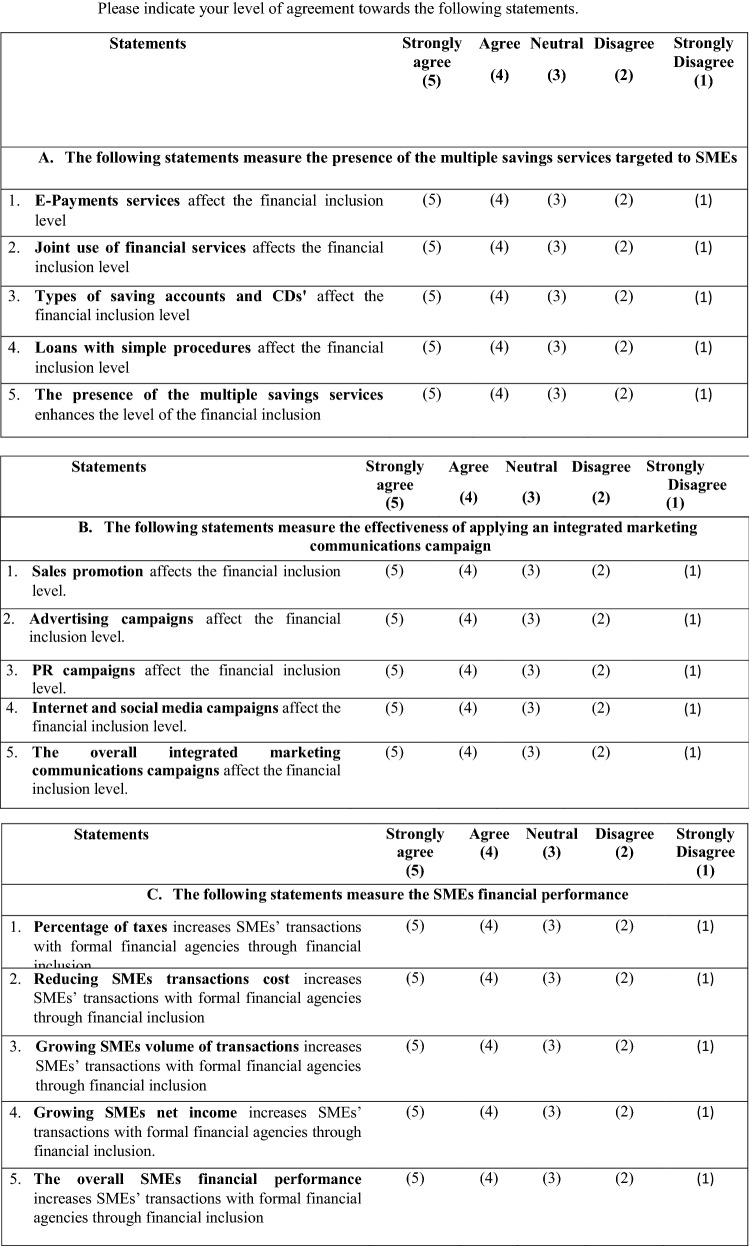

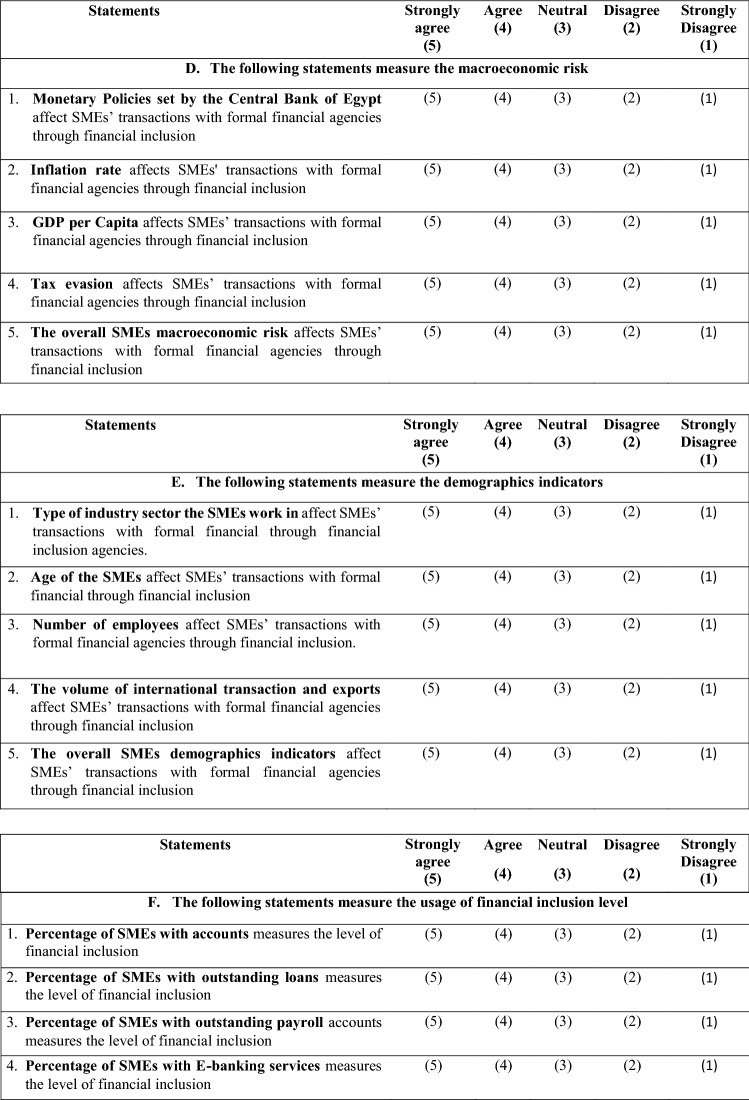

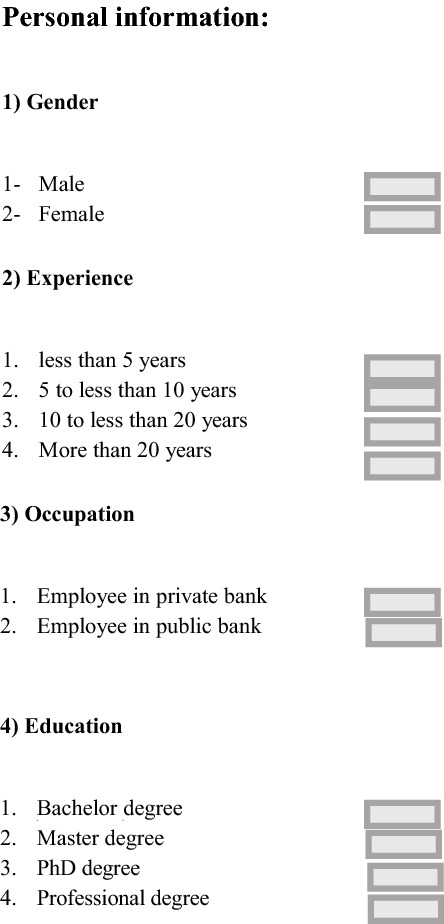
Questionnaire

## Data Availability

The authors declare they have full access to all study data, take full responsibility for the accuracy of the data analysis and have authority over manuscript preparation and decisions to submit the manuscript for publication.

## Appendix

See Fig. 3 and Tables 7, 8.

**Table 7:** Descriptive statistics for some categories of questionnaire’s variables *Source*: Questionnaire data

	N	Minimum	Maximum	Mean	Std. deviation
Access: multiple saving services	355	1	5	3.94	.654
Access: integrated marketing campaign	355	1	5	4.03	.682
Size of financial transactions by SMEs	355	1	5	3.92	.721
Macroeconomic risk factors	355	1	5	4.22	.786
SMEs' demographics	355	1	5	3.96	.673
Usage of financial services	355	1	5	3.90	.659
Valid N (listwise)	355				

## Abbreviations

SMEs: Small- and medium-sized enterprises
PCA: Principal component analysis
SDGs: Sustainable development goals
IFC: International financial access
OECD: Organization for Economic Co-operation and Development
MSME: Ministry of Micro-, Small- and Medium-sized Enterprises
CBE: Central Bank of Egypt
IMT: International money transfers
M2M: Merchant-to-merchant
P2P: Person-to-person
P2M: Person-to-merchant
VCN: Virtual card number
GDP: Growth domestic product
MENAP: Middle East North Africa, Afghanistan and Pakistan

## Footnotes

1 Small- and medium-sized enterprises are businesses in which one, two or three individuals make most of the decisions. There is no serious distinction between private and business assets, and it is subjective to personal factors which play a considerable role in the decision-making process. SME entrepreneurs have much larger stakes in their business than corporate executives do in their corporation. Most countries apply three measures to distinguish SMEs from other companies: capital investment in plant and machinery, number of employees and production volume or turnover [5]. Based on OECD definition, a small- and medium-sized enterprise is defined as the enterprise whose number of employees should not exceed 10–49 employees for small-sized enterprises and balance sheets of EUR 10 million. As for medium-sized enterprises the range is from 50 to 29 employees and balance sheets of EUR 50 million [43, p.17].

2 Age of the firm in this context refers to the number of years of its existence, operations and establishment. Usually, the more experienced firms operating for longer years will have a more creditworthy reputation and history [40, 46].

3 The indicators testing for the level of financial inclusion were derived from the literature and all included on the G section of the questionnaire in Appendix, specifying measurement of financial inclusion level for SMEs, and they are estimated in categories: (1) percentage of financial services, products and infrastructural capital (provided through banks ATM cash-in/cash-out for mobile wallets, person-to-person (P2P), person-to-merchant (P2M), merchant-to-merchant (M2M), international money transfers (IMT), virtual card number); (2) % use of e-payment services; (3) percentage of coverage they receive from being financially included in the banking system; (4) their willingness to switch a percentage of their transactions to cashless payments; (5) awareness and promotion they receive on the financial services; and (6) optimal setting of the monetary policy reflected through the percentage of outstanding loans and payrolls of employees [20, 26, 30, 32, 37, 41, 44, 48, 55, 60].

4 Eigenvalue-based criteria: They explain the variance of data derived from the questionnaire and how the magnitude of these data changes when the principal component analysis is applied, which is basically rotating the data on the plain until the most variability and change is driven from the new rotated eigenvectors and it provides a new intuitive way to justify and explain data variability [8].
